# Carbohydrate fatty acid monosulphate ester adjuvant enhances the immunogenicity of influenza antigens *via* TLR4/2-dependent mechanisms

**DOI:** 10.3389/fimmu.2026.1787181

**Published:** 2026-03-04

**Authors:** Sruthi Vijaya Retnakumar, Suraj Chandrabhan Singh, Srinivasa Reddy Bonam, Camille Chauvin, Mano Joseph Mathew, Ida Busch Nielsen, Christine Boyle, Luuk Hilgers, Max Søgaard, Peter Paul Platenburg, Jagadeesh Bayry

**Affiliations:** 1 Institut National de la Santé et de la Recherche Médicale, Centre de Recherche des Cordeliers, Sorbonne Université, Université Paris Cité, Paris, France; 2 Department of Biological Sciences & Engineering, Indian Institute of Technology Palakkad, Palakkad, India; 3 Vaccine Immunology Laboratory, Department of Applied Biology, CSIR-Indian Institute of Chemical Technology, Hyderabad, India; 4 Academy of Scientific and Innovative Research (AcSIR), Ghaziabad, India; 5 EFREI Research Lab, Panthéon Assas University, Villejuif, France; 6 Laboratoire Génomique, Bioinformatique et Chimie Moléculaire, EA7528, Conservatoire National des Arts et Métiers, HESAM Université, Paris, France; 7 ExpreS2ion Biotechnologies, SCION-DTU Science Park, Hørsholm, Denmark; 8 EpiVax, Providence, RI, United States; 9 LiteVax B.V, Oss, Netherlands

**Keywords:** adjuvants, carbohydrate fatty acid monosulphate ester, dendritic cells, influenza, T cells, toll-like receptors, vaccines

## Abstract

**Introduction:**

Subunit influenza vaccines require potent adjuvants to elicit robust and broad immune responses, particularly against emerging pandemic strains such as H7N9. However, currently approved adjuvants often fail to induce durable and broadly protective immunity. Carbohydrate fatty acid monosulphate ester (CMS), a synthetic glycolipid incorporated into a squalane-in-water emulsion, has demonstrated promising immunostimulatory properties and is currently undergoing phase I clinical evaluation. However, the molecular mechanisms underlying its adjuvanticity in human immune cells remain incompletely understood. We therefore investigated the immunological and molecular mechanisms by which CMS enhances influenza hemagglutinin (HA) immunogenicity.

**Methods:**

Human peripheral blood mononuclear cells (PBMCs) and monocyte-derived dendritic cells (DCs) from healthy donors were stimulated with influenza HA antigen alone or in combination with CMS. Antigen-specific T cell responses were assessed using activation-induced marker assays and intracellular cytokine staining. DC maturation markers and cytokine secretion were analyzed by flow cytometry and ELISA. Antigen uptake was evaluated by fluorescence microscopy and flow cytometry. Bulk RNA sequencing of CMS-stimulated DCs was performed to identify differentially expressed genes and enriched pathways. Toll-like receptor (TLR) involvement was validated using THP-1 reporter assays and pharmacological inhibition in DCs.

**Results:**

CMS significantly enhanced antigen-specific CD4⁺ and CD8⁺ T cell responses in PBMCs. While recombinant H7N9 HA alone poorly activated DCs, co-formulation with CMS induced robust upregulation of co-stimulatory molecules and pro-inflammatory cytokines, leading to a polyfunctional T helper cell response. Transcriptomic profiling revealed strong enrichment of TLR, NF-κB, JAK-STAT, and interferon signaling pathways. Functional studies confirmed that CMS-induced activation depends on TLR4 and TLR2 engagement.

**Discussion:**

CMS enhances influenza antigen immunogenicity by promoting TLR4/2-dependent DC activation, inflammatory signaling, and improved antigen presentation. These findings define the mechanistic basis of CMS adjuvanticity and support its development as a promising adjuvant platform for next-generation influenza vaccines targeting emerging pandemic strains.

## 1 Introduction

Despite widespread annual vaccination campaigns, influenza viruses continue to impose a significant public health burden, causing an estimated 290,000 to 650,000 deaths globally each year due to seasonal infections (1, 2). Multiple influenza vaccine strategies have been developed and routinely updated to match emerging viral strains; however, their overall effectiveness remains below 40% in the general population (3–5). The effectiveness is further diminished in susceptible populations, including children, older adults, and other immunocompromised individuals with chronic diseases, who are at greater risk of developing severe illnesses and complications due to influenza infection (6, 7). The emergence of novel pandemic influenza strains, generated through antigenic reassortment, poses a further substantial threat to global health due to the absence of pre‐existing antibodies or memory T cells (8). The COVID-19 pandemic heightened the global urgency of enhanced vaccine development, invigorating efforts to develop broadly protective next-generation influenza vaccines.

Influenza vaccine platforms have evolved substantially from traditional egg-based, inactivated virus formulations to highly adaptable recombinant protein and mRNA-based technologies, each offering distinct advantages in pandemic preparedness and public health impact. Next-generation mRNA-based influenza vaccines are currently undergoing clinical trials and have demonstrated superior efficacy compared to licensed vaccines (9–11). Nevertheless, recombinant subunit vaccines remain an important complementary approach due to their well-established, predictable safety profiles with less systemic reactogenicity and rare adverse events compared to mRNA/LNP vaccines, which can occasionally provoke stronger innate responses, fever, and rarely myocarditis (11–14). Most recombinant subunit influenza vaccines utilize the trimeric surface glycoprotein hemagglutinin (HA), by which the virus attaches to sialic acid-containing proteins on the host cell surface, and therefore a key determinant of virulence (15, 16). However, purified subunit vaccines are inherently less immunogenic, lacking pathogen-associated molecular patterns (PAMPs), and therefore, adjuvants have become an inevitable component in their formulations.

In addition to lowering the required vaccine antigen doses, adjuvants have a crucial role in shaping the resulting immune response by generating a mixed T helper cell response (Th1/Th2), thereby driving potent cellular and humoral responses. Currently licensed influenza vaccines typically employ alum or squalene-in-water (S/W) emulsion adjuvants such as MF59 and AS03 (3, 17, 18). Although alum is widely used, its limited efficacy and potential reactogenicity have been documented in pandemic influenza vaccines against H1N1 and H5N1 strains (19, 20). In contrast, currently approved S/W emulsions have demonstrated favorable safety profiles alongside enhanced immunogenicity; MF59 and AS03 have been successfully deployed in both seasonal and pandemic vaccines, eliciting stronger and broader immune responses against both homologous and heterologous influenza strains across general and high-risk populations (21, 22).

A squalane-in-water emulsion LiteVax™ adjuvant incorporating a synthetic carbohydrate fatty acid monosulphate ester (CMS) represents a promising candidate, currently advancing through phase I clinical trials for a licensed seasonal influenza vaccine (23–25). Beyond serving as an antigen delivery vehicle, the immunostimulatory properties of its components, including CMS, have been evaluated. Our previous studies have demonstrated that CMS robustly activates human antigen-presenting cells (APCs), with CMS-treated dendritic cells (DCs) capable of polarizing robust T helper responses (26). However, the nature of antigen-specific cellular responses generated by the adjuvant to influenza antigens and the precise receptors and molecular pathways engaged by CMS to activate APCs remain to be investigated.

Classically, only H1, H2, and H3 influenza A subtypes have been responsible for human pandemics. However, the emergence and spread of highly pathogenic H5N1 and H7N9 avian influenza viruses, both associated with severe human disease and high case-fatality rates, have raised substantial concern about their pandemic potential. Although recent H5N1 infections, particularly those caused by Clade 2.3.4.4b viruses, are receiving increased attention (27, 28), H7N9 has historically ranked among the highest-risk viruses in the United States Centers for Disease Control and Prevention’s Influenza Risk Assessment Tool because of its capacity to infect humans, cause severe illness, and persist in avian reservoirs (29). To date, there is no universally licensed, widely deployed H7N9 vaccine for humans, and available pre-pandemic candidates are generally poorly immunogenic without strong adjuvants, often requiring high antigen doses and multiple administrations while offering limited breadth against antigenically drifted variants and suboptimal durability of protection (30, 31). These limitations underscore an urgent need for improved, adjuvanted H7N9 vaccine formulations capable of inducing robust, long-lasting, and broadly protective immunity.

In this study, we investigate the immunomodulatory effects of the CMS adjuvant in human immune cells, with an emphasis on its capacity to enhance the immunogenicity of influenza HA antigens. We report that CMS robustly increases antigen-specific T cell responses to H1N1 HA peptide pools in human peripheral blood mononuclear cells (PBMCs). Although recombinant H7N9 HA antigens alone induced weak immunogenicity in DCs, co-formulation with CMS substantially boosted their immunogenic potential. We also examined the T-cell polarizing properties of this adjuvant formulation. Mechanistically, our data show that CMS triggers innate immunity *via* toll-like receptor (TLR)-mediated inflammatory signaling and promotes improved antigen presentation to T cells. Together, our findings indicate that combining recombinant HA proteins with the CMS platform may represent an effective future vaccine approach for emerging pandemic influenza strains.

## 2 Materials and methods

### 2.1 Recombinant protein expression and purification

The H7N9 (A/Anhui/1/2013) HA gene was cloned into the pExpreS2–1 vector (ExpreS2ion Biotechnologies, Hørsholm, Denmark) and transfected into Drosophila S2 insect cells. Stable cell lines were established in three weeks in T-flask culture, which were then inoculated at 8 x 10^6^ cells/ml in a shake flask and harvested after 3–4 days. A 3.8 L harvest was thawed and processed by tangential flow filtration (TFF), ultrafiltered to 250 ml, and subsequently diafiltered with 7.5X turn over volumes into a buffer containing 20mM Tris pH 7.5, 100mM NaCl. The protein was captured from the load sample on 2ml CaptureSelect C-tagXL. The protein was eluted with increasing concentrations of MgCl_2_ (250mM, 500mM, 1M, and 2M). Flowthrough was reapplied to the column until the protein of interest was depleted. Eluates from each elution concentration were analyzed using SDS-PAGE, and the fractions containing the protein were pooled and concentrated on a 10kDa cutoff spin filter. For further purification, a Superdex 200pg 26/600 column was used for size exclusion chromatography (SEC). Peak fractions from the SEC run were determined in protein size on analytical SEC (Superdex200 Increase 3.2/300). The peak containing protein of the correct size was pooled and concentrated on a 10kDa cutoff spinfilter (**Supplementary Figure 1**).

### 2.2 Isolation of PBMCs, generation of DCs

Buffy coats of healthy donors obtained from Etablissement Français du Sang, Rungis, France (Institut National de la Santé et de la Recherche-EFS ethical committee convention 18/EFS/033) were subjected to Ficoll density gradient centrifugation to isolate PBMCs. Monocytes were positively selected using CD14 Microbeads (Miltenyi Biotec) and cultured in the presence of granulocyte-macrophage colony-stimulating factor (GM-CSF, 1000 IU/10^6^ cells) (Miltenyi Biotec) and IL-4 (500 IU/10^6^ cells) (Miltenyi Biotec) in RPMI-1640 supplemented with 10% fetal bovine serum (FBS) and 1% penicillin-streptomycin for 5 days to generate immature DCs.

### 2.3 Treatment of PBMCs with the antigen or adjuvant for activation-induced marker assay

CMS was provided by LiteVax BV, Netherlands. Isolated human PBMCs were seeded at a density of 1.5 x 10^6^ cells/well in a 48-well plate in 300 μl of RPMI supplemented with 5% human AB serum, 100 IU/ml penicillin, and 100 µg/ml streptomycin. The cells were treated with H1N1 HA peptide pools (Miltenyi Biotec, 130-099-803) at a concentration of 0.5 μg/ml alone or combined with CMS (125 μg/ml) for 6 days. On days 2 and 4, cultures were supplemented with 10 ng/ml IL-2 by half medium replacement. On day 6, the cells were harvested by centrifugation and resuspended in fresh medium in a 96-well plate. The cells were restimulated with H1N1 peptide pools at a concentration of 1 μg/ml for 16 hrs in the presence of CD40 monoclonal antibody (1 μg/ml, clone: HB14; Miltenyi Biotec) and CD28 monoclonal antibody (1 μg/ml, clone: 37407; R&D Systems). Following restimulation, cells were stained for surface activation markers and analyzed by flow cytometry.

### 2.4 Treatment of DCs with the antigen or adjuvant

Immature DCs were seeded at a density of 0.5 x 10^6^ cells/ml/well in a 24-well plate in culture media supplemented with GM-CSF and IL-4, and left untreated (cells alone) or stimulated with recombinant H7N9 HA antigen (10 µg/ml) alone or combined with CMS (125 µg/ml) for the indicated time points. For TLR signaling inhibition, DCs were pre-incubated with CLI095 (3 µM), TL2-C29 (200 µM), CU-CPT9a (10 µM) (all from Invivogen) or 0.1% dimethyl sulfoxide prior to CMS stimulation (125 µg/ml). Post-treatment, culture supernatants were collected for cytokine quantification by ELISA, and cells were analyzed for phenotypic markers by flow cytometry.

### 2.5 Microscopy analysis of adjuvant-antigen association and endocytosis

Recombinant H7N9 HA protein was fluorescently labeled with Alexa Fluor™ 488 Microscale Protein Labelling Kit (Invitrogen, A30006) following the manufacturer’s instructions. CMS was mixed with 100 μM Nile red (Invitrogen, N1142) and incubated for 30 min at room temperature. The colloidal CMS particles were pelleted by centrifugation at 5000 rpm, and excess stain was removed by washing 3 times with 1X PBS. The particles were resuspended in an appropriate volume of 1X PBS and mixed with labeled HA protein. The mixture was added to 100,000 DCs in 100 μl culture medium in a 96-well plate at final concentrations of 125 μg/ml CMS and 5 μg/ml HA. The cells were imaged under a fluorescence microscope after 1 hr. To quantitatively measure antigen endocytosis, samples were acquired and analyzed by flow cytometry.

### 2.6 Co-culture of DCs and CD4^+^ T cells

Followed by treatment with the antigens and the adjuvants as described above, the DCs were co-cultured with autologous CD4^+^ T cells to analyze the T cell polarization. Autologous total CD4^+^ T cells were isolated from PBMCs using magnetic bead isolation kits (Miltenyi Biotec) and cultured along with the stimulated DCs at a ratio of 10:1 (T cell to DCs) for 5 days in serum-free X-VIVO medium (Lonza). After co-culture, the cells were washed and stimulated with phorbol 12-myristate 13-acetate (50 ng/ml/0.5 x 10^6^ cells) and ionomycin (500 ng/ml/0.5 x 10^6^ cells) (Sigma–Aldrich), along with GolgiStop (BD Biosciences) for 4 hrs. After staining the cells for the surface marker CD4, and CD127, fixation/permeabilization was carried out with an intracellular fixation & permeabilization buffer (eBioscience) for intracellular staining of IFN-γ, IL-17A, IL-4, FoxP3 to determine the frequency of Th1, Th2, Th17, and Treg subpopulations by flow cytometry.

### 2.7 THP-1 cell stimulation

THP1-Dual, THP1-Dual MD2-CD14-TLR4, and THP1-Dual KO-TLR4 reporter cells (Invivogen) were resuspended at a concentration of 100,000 cells per well in a 96-well U-bottom plate in 200 µl RPMI 1640 Medium, GlutaMAX Supplement (Gibco), supplemented with 25mM HEPES, 10% FBS, 100 IU/ml penicillin, and 100 µg/ml streptomycin. Cells were incubated for 20 hrs at 37 °C in a humidified incubator at 5% CO_2_ with the indicated treatments. To assess NF-κB activity *via* SEAP reporter activity, 20 µl of cell-free supernatants were combined with 180 µl QUANTI-Blue solution (Invivogen) in a clear 96-well flat-bottom plate and incubated for 3.5 to 4 hrs at 37 °C. Optical density was read at 620 nm using a microplate reader (TECAN).

### 2.8 Endotoxin quantification

Endotoxin levels in recombinant H7N9 HA antigen and CMS adjuvant were determined using the Limulus Amebocyte Lysate (LAL) test (Pierce™ Chromogenic Endotoxin Quantitation Kit, Thermo Scientific) according to the manufacturer’s protocol.

### 2.9 Flow cytometry

The following antibodies were used for flow cytometry analysis of various surface/intracellular markers. BD Biosciences: CD80-AF700 (clone: L307.4), CD86-FITC (clone: FUN-1), CD54-APC (clone: HA58), CD83-APC (clone: HB15e), HLA-DR-PerCP-Cy5.5 (clone: G46-6), CD8-Pacific Blue (clone: RPA-T8), IFN-γ-FITC (clone: 4S.B3), IL-4-PE (clone: MP4-25D2); Beckman Coulter: CD40-PE (clone: MAB89); BioLegend: CD4-PerCP (clone: SK3); Invitrogen: CD3-FITC (clone: 7D6); eBiosciences: FoxP3-APC (clone: 236A/E7), IL-17A-PE (clone: eBio64cap17); Miltenyi Biotec: CD154-APC (clone: 5C8), CD137-PE (clone: 4B4-1), TNF-α-APC (clone: cA2). The experiments were performed using LSR II flow cytometer (BD Biosciences), and the data were analyzed by BD FACS DIVA and FlowJo software.

### 2.10 ELISA

The cytokines (IL-1β, IL-6, IL-8, IL-10, IL-12, and TNF-α) in the cell-free culture supernatants were quantified using commercial ELISA kits (Invitrogen).

### 2.11 RNA sequencing

Monocyte-derived DCs from healthy donors were treated under different conditions and then subjected to bulk RNA sequencing to assess the changes in mRNA expression. The study included two experimental groups: control (n = 3 donors) and CMS adjuvant-treated DCs (n = 3 donors). Total RNA was extracted from each sample using a standard protocol, followed by a quality assessment with an Agilent Bioanalyzer. Library preparation was performed using an RNA-seq kit, and sequencing was conducted on an Illumina platform to generate high-quality paired-end reads. The raw data supporting this study have been deposited in the Gene Expression Omnibus (GEO) Database under the accession number GSE318332.

### 2.12 Data pre-processing and normalization

Raw sequencing reads were processed using FastQC (v0.12.1) for quality control, followed by trimming with Trimmomatic (v0.39) to remove adapter sequences and low-quality bases. STAR (v2.7.11b) was used to align the cleaned reads to the reference genome (GRCh38), and HTSeq-count (v2.0.5) was used to measure the number of genes that were expressed at the expression level.

### 2.13 Differential expression analysis

Differential gene expression analysis was performed using DESeq2 (32) to compare gene expression levels between treatment groups and control samples. Genes with an adjusted p-value (Benjamini-Hochberg correction) < 0.05 and log2 fold change > 1 were considered significantly differentially expressed.

### 2.14 Pathway enrichment analysis

The top 200 differentially expressed genes upregulated in the CMS treatment condition compared to the control were subjected to functional enrichment analysis using KEGG (Release 117.0) and Reactome pathway databases (v94) or associated reference. Over-representation analysis was conducted using the R (v4.4.2) package clusterProfiler (v4.14.6) (33) to identify enriched pathways in these databases.

### 2.15 Protein-protein interaction network construction and hub genes identification

The PPI network was constructed using the STRINGdb package (v2.18.0) (34) in R. The list of top 200 DEGs was mapped to their corresponding STRING identifiers using the built-in human protein database (STRING V12.0). The PPI network was visualized and analyzed using the igraph package. Genes with the highest degree values were considered as hub genes, reflecting their potential as a central regulator of protein interaction networks.

### 2.16 Statistical analysis

Statistical analyses were performed by one-way ANOVA with Tukey’s multiple comparison post-test, or two-way Mann–Whitney U test, and P < 0.05 was considered significant. The analyses were performed using Prism 8 (GraphPad Software Inc., CA).

## 3 Results

### 3.1 CMS adjuvant enhances antigen-specific T cell responses to influenza HA antigens in human PBMCs

**Figure 1:**
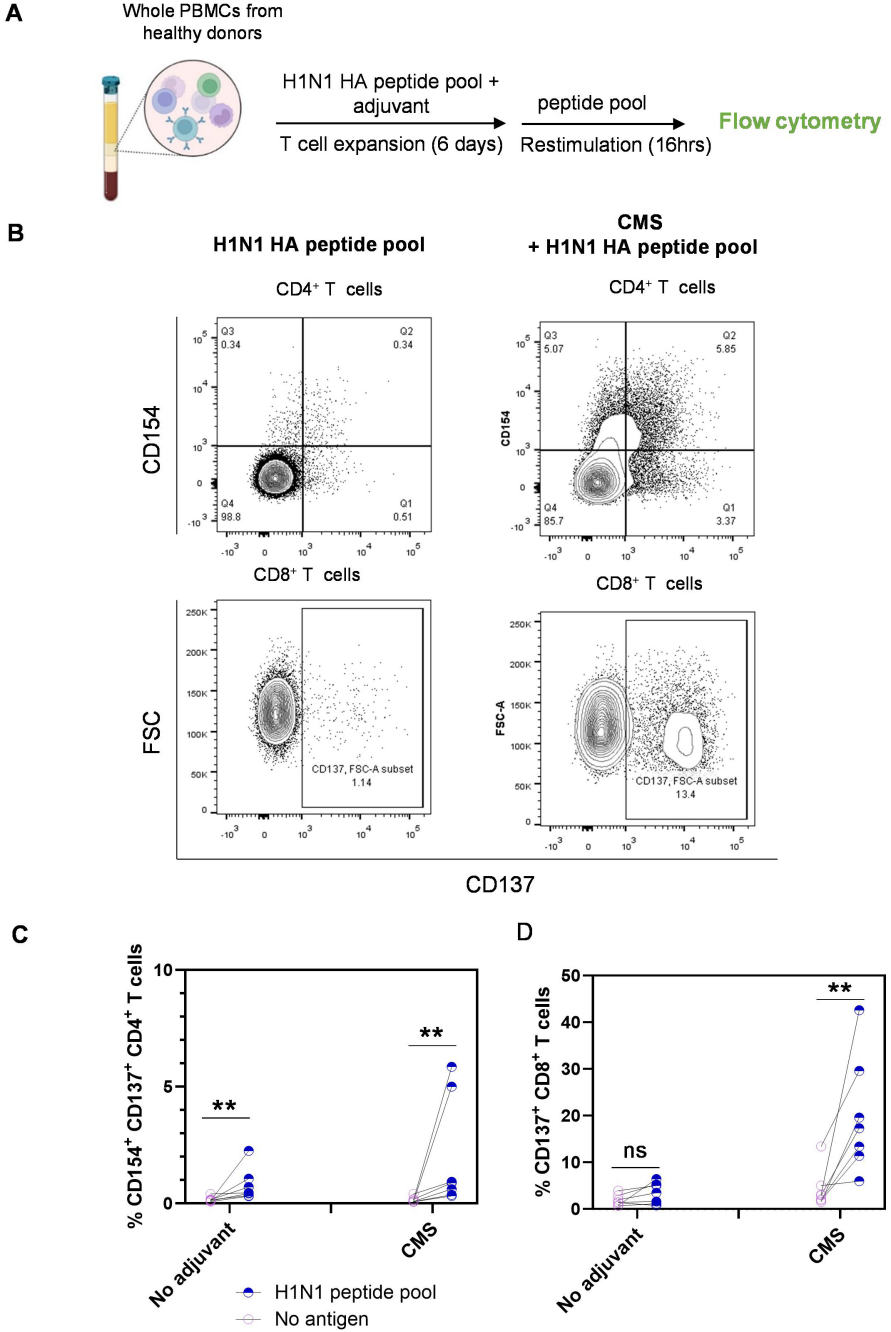
CMS adjuvant enhances antigen-specific T cell responses to H1N1 HA peptide pools in human PBMCs. **(A)** Scheme of the AIM assay setup. PBMCs from healthy donors were treated with H1N1 HA peptide pools alone or in combination with the adjuvant for 6 days and restimulated with peptide pools for 16 hrs in the presence of anti-CD40 and anti-CD28, followed by surface staining and flow cytometry analysis of AIMs. **(B)** Representative dot plots showing frequencies of CD154^+^ CD137^+^ CD4^+^ and CD137^+^CD8^+^ T cells for the indicated treatments. **(C)** Summarized frequencies of HA-specific CD154^+^ CD137^+^ CD4^+^ and CD137^+^ CD8^+^ T cells from independent donors (n=9). Statistics are **P < 0.01; ns, not significant as determined by Mann-Whitney’s U test **(C, D)**. AIM, activation-induced marker; SD, standard deviation.

Adjuvant enhances the immunogenicity of antigens through various mechanisms. To understand the cellular mechanisms by which CMS enhances the immunogenicity of influenza HA antigens, we have set up activation-induced marker (AIM) assays with human PBMCs from healthy donors. PBMCs were treated with H1N1 HA peptide pools either alone or in combination with CMS for 6 days and then restimulated with the peptide pools for 16 hrs, followed by flow cytometry analysis of AIMs, such as CD154 and CD137 (**Figures 1A, B**). Although peptide pools alone induced significant antigen-specific CD4^+^ T cell responses, the presence of the adjuvant further increased the frequency of responding cells (**Figure 1C**). Notably, the peptide pools alone were unable to efficiently induce CD8^+^ T cell responses, whereas combining with CMS significantly enhanced the frequency of CD137^+^ CD8^+^ T cells (**Figure 1D**).

### 3.2 CMS enhances the antigenicity of recombinant H7N9 HA protein on human DCs

Maturation of DCs following antigen uptake represents a key step in initiating innate and adaptive immune responses to vaccines. Subunit vaccine antigens, such as recombinant proteins, often show limited capacity to activate DCs due to the absence of PAMPs typical of whole viruses (35). We have previously demonstrated that CMS exerts a strong immunostimulatory effect on DCs (26). The inherent ability of CMS in the commercial squalene-based emulsion to markedly enhance antigen-specific T cell responses to classical pandemic strains prompted us to investigate the combination of CMS with a recombinant H7N9 HA protein (potential pandemic strain) to assess its immunogenic potential on human DCs.

Monocytes isolated from buffy coats of healthy donors were differentiated into DCs and treated with the antigen alone or in combination with CMS for 48 hrs. The H7N9 HA protein did not promote significant enhancement in the expression of DC-activation markers compared to unstimulated cells, confirming its poor intrinsic immunostimulatory capacity.

**Figure 2:**
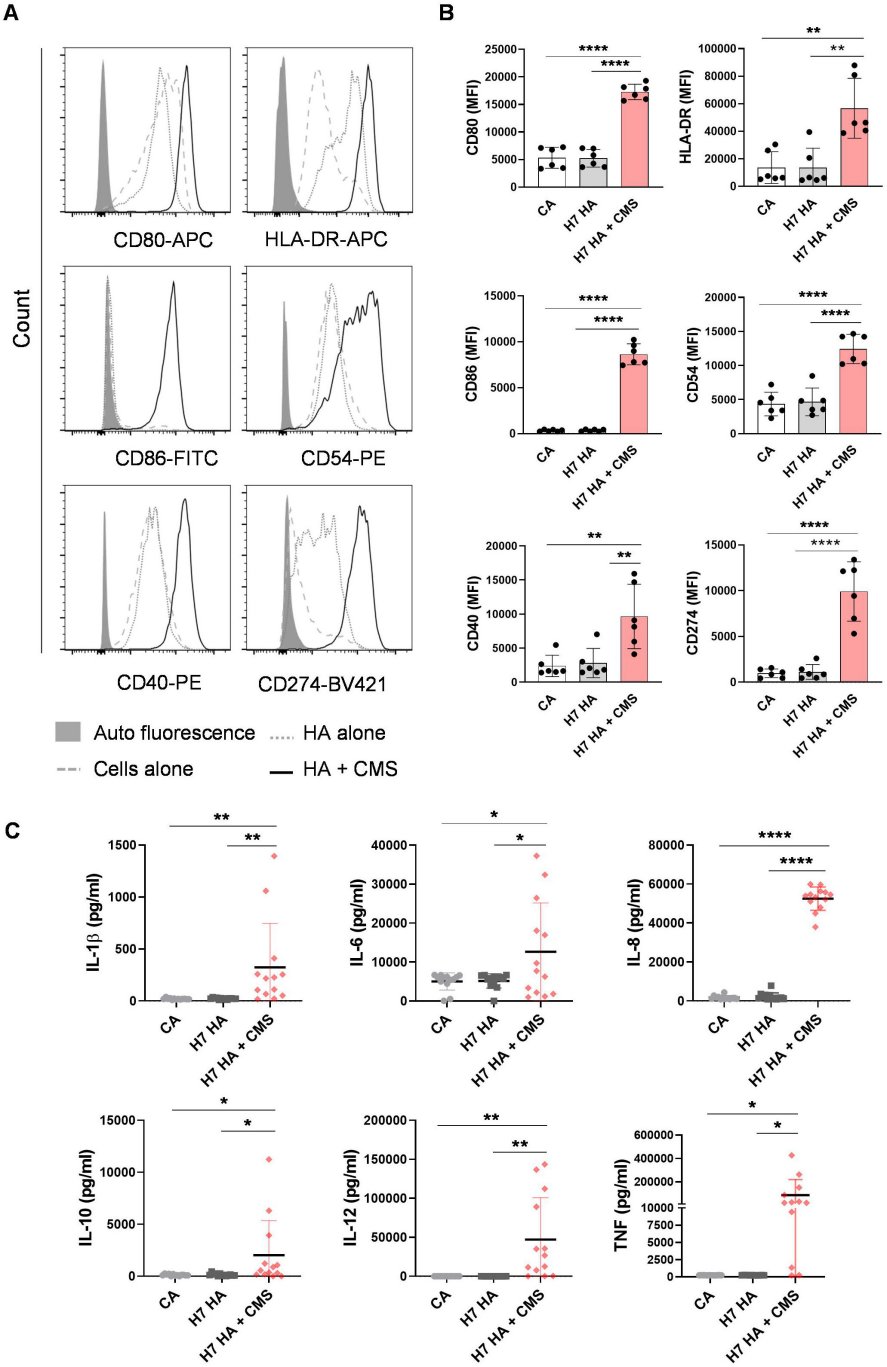
CMS enhances the immunogenicity of H7N9 HA protein antigen on human DCs. Immature DCs were left alone or treated with H7N9 HA (10 μg/ml) antigen alone or antigen + CMS (125 µg/ml) for 48 h. After incubation, cells were subjected to surface phenotyping by FACS. **(A, B**) Representative histograms and scatter plots representing the mean ± SD (n = 6 donors) values of expression (median fluorescence intensities, MFI) of CD80, CD86, CD40, HLA-DR, CD54, and CD274. **(C)** Cell-free supernatants were collected and analyzed for cytokines. Amount of secretion (mean ± SD, n = 12-13) of IL-1β, IL-6, IL-8, IL-10, IL-12p70 and TNF-α, (all in pg/ml) (n = 10–13 donors). Statistics are *P < 0.05; **P < 0.01; ****P < 0.0001 as determined by one-way ANOVA with Tukey’s multiple comparisons post-test **(B, C)**. SD, standard deviation.

In line with previous results, the stimulation of DCs with HA in the presence of CMS led to an increased upregulation of various markers associated with activation of DCs such as CD80, CD86, CD40, CD274, CD54, and HLA-DR, compared to unadjuvanted antigen (**Figures 2A, B**). Moreover, measured endotoxin levels were ~0.005 EU/mL for HA and ~0.01 EU/mL for CMS at their maximum working concentrations (10 µg/mL HA; 250 µg/mL CMS). These concentrations were used in DCs and subsequent THP-1 assays. The endotoxin levels remained well below levels known to trigger innate immune activation.

Activated DCs secrete immunomodulatory cytokines that have a critical role in signaling the polarization of effector T helper cell responses. Cytokine production in supernatants from antigen- and/or adjuvant-treated DCs was measured by ELISA. While the antigen alone did not induce any cytokine responses, co-treatment with CMS triggered a higher secretion of the pro-inflammatory cytokines IL-1β, IL-6, IL-8, TNF, IL-12, as well as the anti-inflammatory cytokine IL-10 (**Figure 2C**). These findings demonstrate that CMS effectively enhances the immunostimulatory capacity of recombinant H7N9 HA protein through potent activation of human DCs.

### 3.3 CMS associates with the H7N9 HA antigen and is endocytosed by DCs

**Figure 3:**
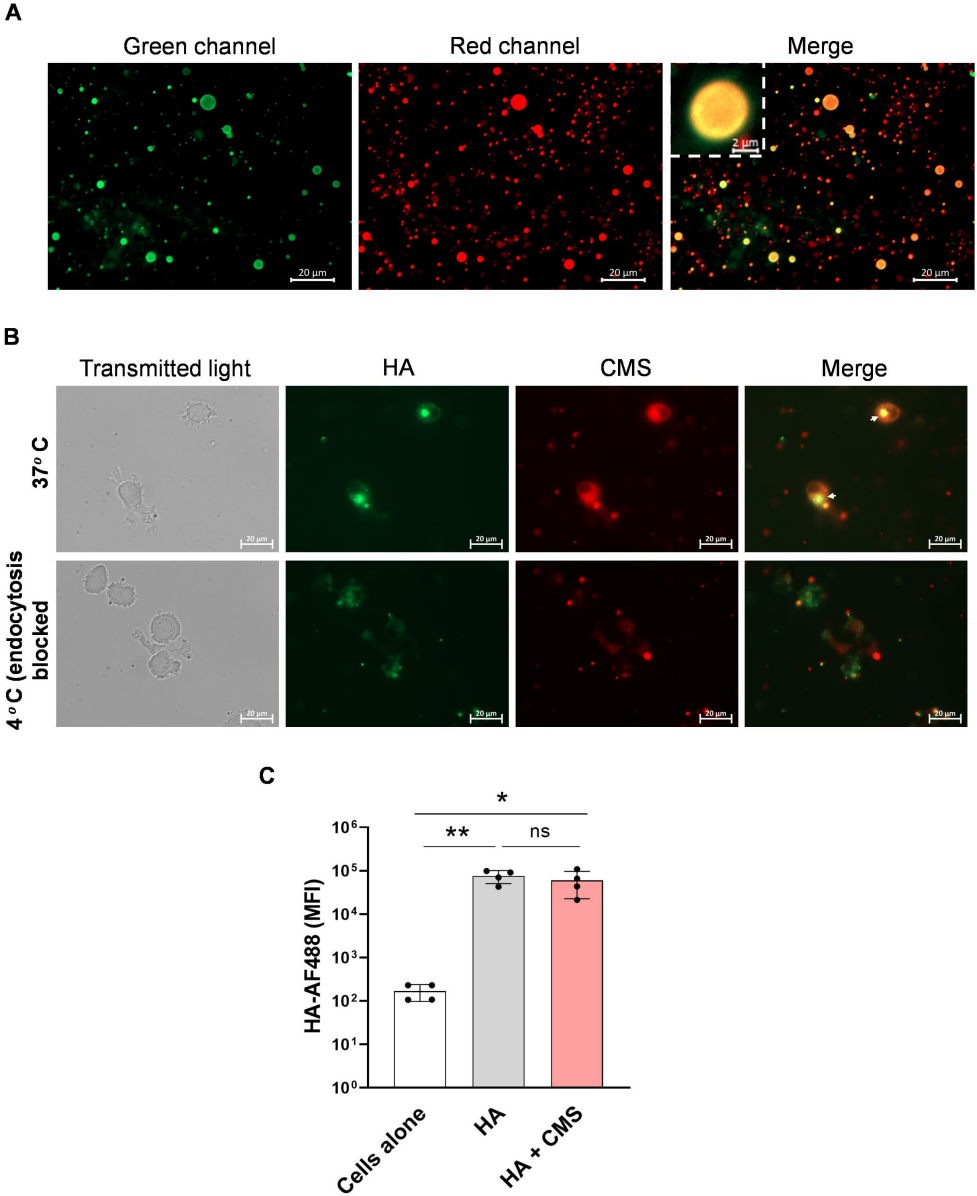
CMS associates with H7N9 HA, and the antigen-adjuvant complex is endocytosed by DCs. **(A)** Fluorescence microscopy image showing the association of HA antigens around CMS droplets. The HA antigen and CMS adjuvant were labeled with AF488 (green) and Nile red (red), respectively. **(B)** Microscopy images showing the endocytosis of labeled antigen-adjuvant complex by DCs at 37°C and 4°C. **(C)** Flow cytometry analysis of antigen uptake, mean ± SD (n = 4 donors) values of median fluorescence intensities (MFI). Statistics are *P < 0.05; **P < 0.01; ns, not significant as determined by one-way ANOVA with Tukey’s multiple comparisons post-test **(C)**. SD, standard deviation.

Adjuvants generally first exert their actions by binding to antigens, and the antigen-adjuvant complex is delivered to APCs, which are then endocytosed by the APCs to form endosomes (36, 37). To visualize the association between CMS and the H7N9 HA protein, the antigen was labeled with Alexa Fluor 488, and the adjuvant was stained with Nile Red, a lipophilic dye that binds to the fatty acid chains of CMS (**Figure 3A**). DCs were incubated with the labeled antigen–adjuvant complex for 1 hr at 37°C and then examined by fluorescence microscopy. The colocalization of the fluorescent signals within the cells indicated association and uptake of the antigen-adjuvant complex by DCs. This process was markedly reduced when the incubation was performed at 4°C, confirming uptake through active endocytosis (**Figure 3B**). However, a quantitative assessment by flow cytometry showed comparable levels of antigen internalization with or without CMS, suggesting that the adjuvant does not significantly alter the overall rate of antigen uptake (**Figure 3C**).

### 3.4 CMS enhances the ability of H7N9 HA antigen-stimulated DCs to polarize T-cell responses

**Figure 4:**
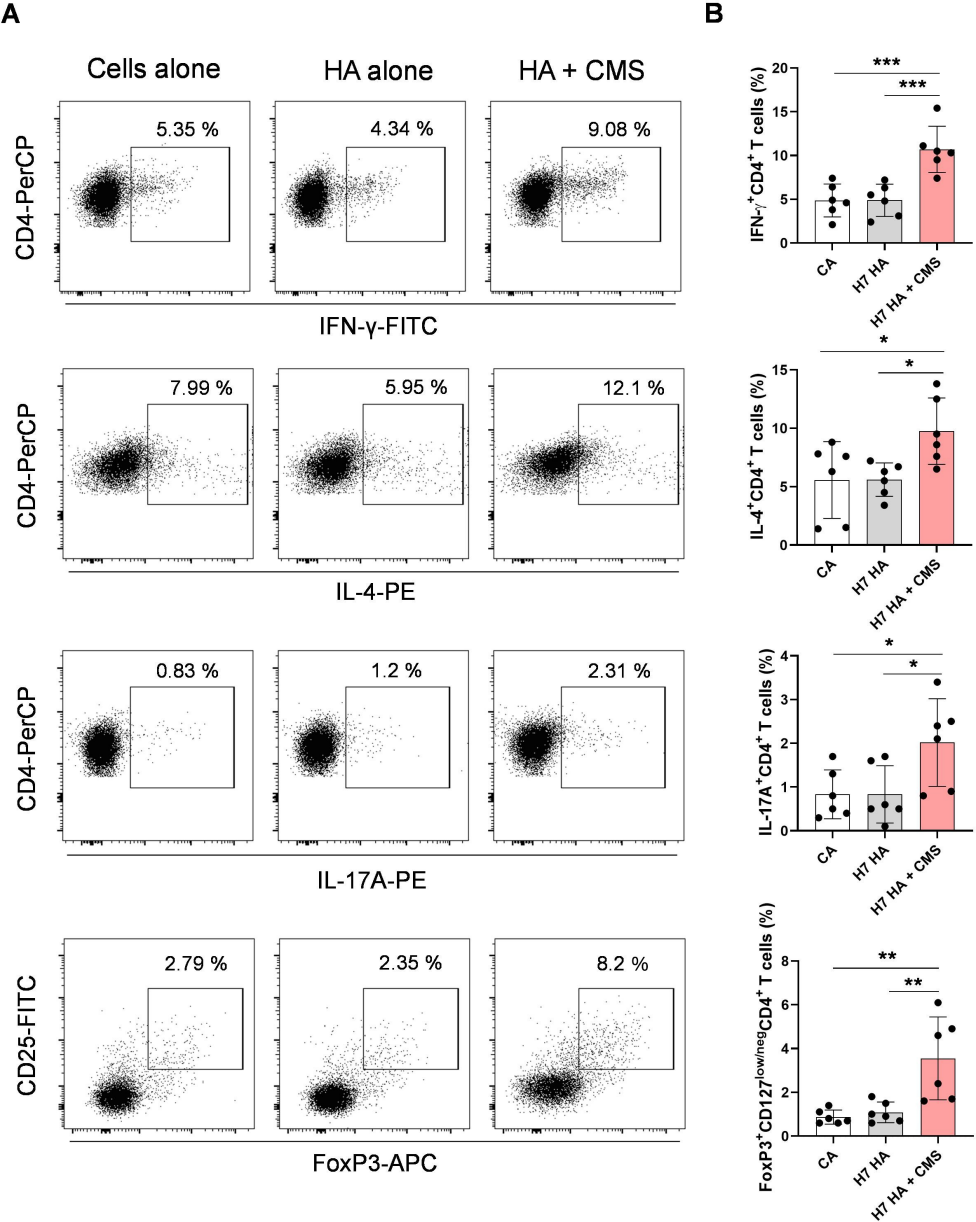
Adjuvant + antigen-stimulated DCs polarize mixed T helper cell responses. Immature DCs were left untreated or treated with H7N9 HA antigen alone (10 μg/ml) or antigen + CMS (125 µg/ml) for 48 (h) After incubation, DCs were washed and co-cultured with purified autologous CD4^+^ total T cells (1:10 ratio) for 5 days. After 5 days, cells were subjected to intracellular cytokine staining for Th1 (IFN-γ^+^CD4^+^), Th2 (IL-4^+^CD4^+^), Th17 (IL-17A^+^CD4^+^), and Tregs (CD25^+^FoxP3^+^CD127^low/neg^ CD4^+^), followed by FACS analysis. **(A, B)** Representative dot-plots and summarized frequencies (mean ± SD, n = 7 donors) are presented. Statistics are *P < 0.05; **P < 0.01; ***P < 0.001; as determined by one-way ANOVA with Tukey’s multiple comparisons post-test **(B)**. CA, cells alone; SD, standard deviation.

We then investigated the capacity of DCs stimulated with adjuvanted H7N9 HA antigen to polarize distinct effector CD4^+^ T cell responses. The stimulated DCs were washed and co-cultured with autologous total CD4^+^ T cells for 5 days, followed by intracellular staining for T cell lineage-specific markers. The flow cytometry analysis revealed an upregulation of Th1 (IFN-γ^+^CD4^+^), Th2 (IL-4^+^CD4^+^), and Th17 (IL-17A^+^CD4^+^) cell responses when DCs stimulated with adjuvanted antigen were co-cultured with total CD4^+^ T cells (**Figures 4A, B**). CD4^+^ T cell responses were lower in co-cultures with unstimulated control DCs and with DCs stimulated with HA antigen alone. In addition, the frequency of FoxP3-expressing CD4^+^ T cells was increased under these conditions (**Figures 4A, B**). These results indicate that DCs stimulated with CMS-adjuvanted influenza antigens have the capacity to induce mixed T helper responses, along with potential Treg induction.

### 3.5 Pathway enrichment analysis of differentially expressed genes in CMS-treated DCs shows activation of TLR-dependent immune response pathways

**Figure 5:**
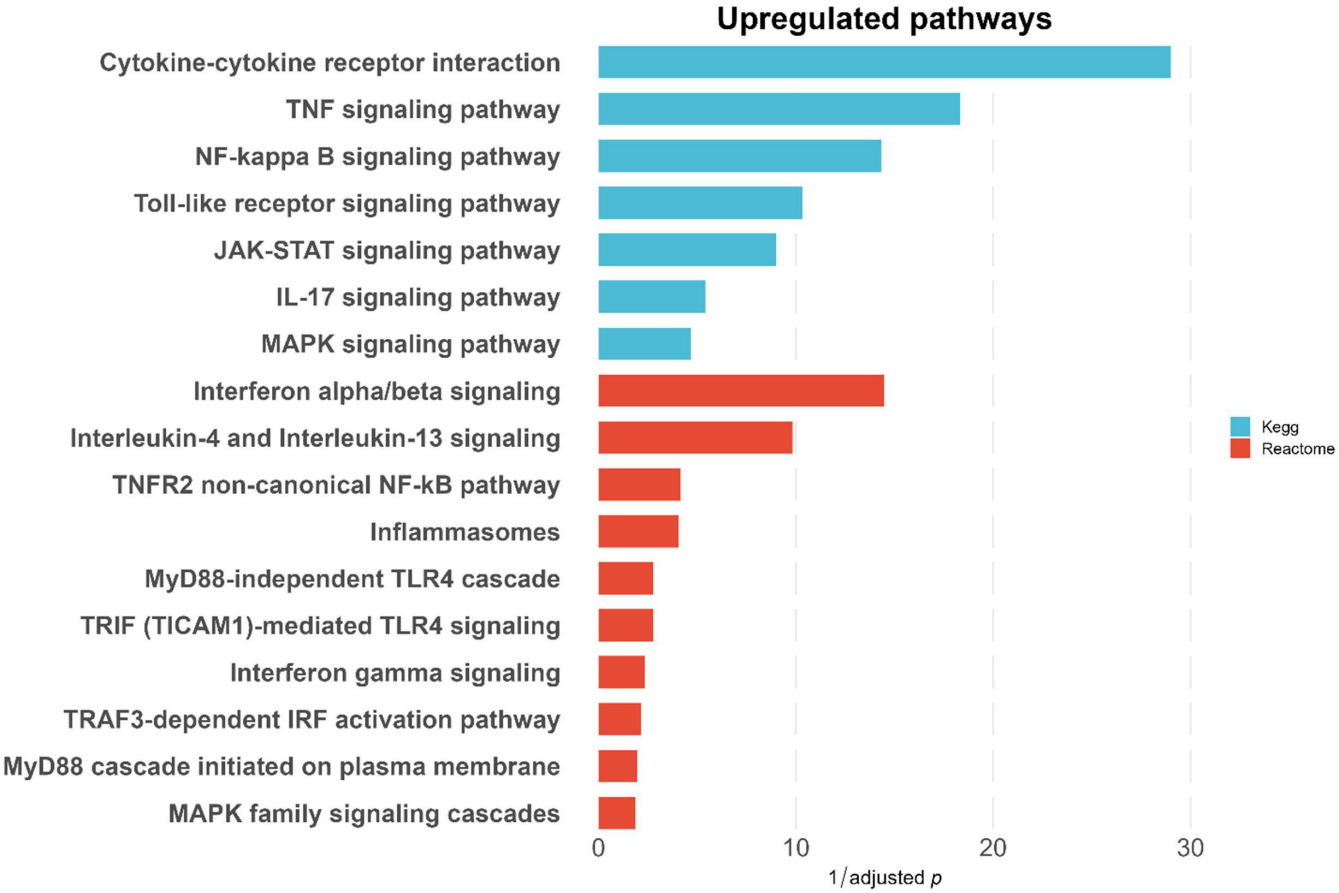
Upregulated immune signaling pathways in CMS-treated DCs. Bar plot showing significantly upregulated pathways in CMS-treated DCs compared to untreated controls, identified using KEGG (blue) and Reactome (red) pathway enrichment analyses. Prominent pathways include cytokine–cytokine receptor interaction, TNF, NF-κB, TLR, and JAK-STAT signaling, as well as interferon α/β and γ signaling cascades. The enrichment of these pathways highlights the strong activation of innate immune and pro-inflammatory signaling, suggesting that CMS effectively stimulates DC activation and cytokine-mediated immune responses.

To elucidate the molecular mechanisms by which CMS activates the APCs and thereby enhances the immunogenicity of influenza HA antigens, RNA-sequence analysis was performed on DCs stimulated with CMS for six hours. Pathway enrichment of 200 upregulated genes indicated robust activation of multiple innate immune and inflammatory cascades (**Figure 5**). The top enriched KEGG pathways included TNF, NF-κB, JAK-STAT, and TLR signaling, consistent with cytokine-driven amplification of immune responses mediated by TLR engagement.

Among Reactome pathways, interferon-α/β and interleukin-4/13 signaling were predominantly upregulated, reflecting activation of both antiviral and Th2-associated immune responses. Additionally, enrichment of MyD88-independent TLR4, TRIF-mediated TLR4, and TRAF3-dependent IRF activation pathways highlighted the engagement of both canonical and non-canonical TLR4 downstream branches. These pathways collectively encompass the MyD88-NF-κB axis driving pro-inflammatory cytokine production and the TRIF-IRF3 axis promoting type I interferon responses.

The selection of the top 200 upregulated genes for the pathway enrichment analysis was based on a balance between biological interpretability and network robustness. Specifically, this threshold captured the most strongly and consistently upregulated transcripts while avoiding the inclusion of weakly expressed or low-confidence genes that can introduce noise into downstream analyses, particularly in PPI network construction (**Supplementary Figure 2A**).

To ensure that this cutoff was not arbitrary, we additionally performed STRING-based protein–protein interaction analyses using the top 300 and 400 upregulated genes (**Supplementary Figures 2B, C**). These analyses showed highly similar network structures, key hub genes, and enriched biological pathways. The 200-gene set represented the minimal and optimal gene subset that retained the core interaction network, whereas larger gene sets mainly added low-connectivity peripheral nodes without changing the overall biological interpretation. Therefore, the top 200 genes were chosen for clarity and interpretability, while the main conclusions remained consistent across different gene cutoffs.

Together, these findings suggest that CMS activates DCs through TLR-dependent mechanisms, resulting in broad stimulation of inflammatory and interferon signaling networks that coordinate cytokine production and immune effector functions.

### 3.6 TLR-driven hub genes and antigen presentation machinery are upregulated in CMS-treated DCs

**Figure 6:**
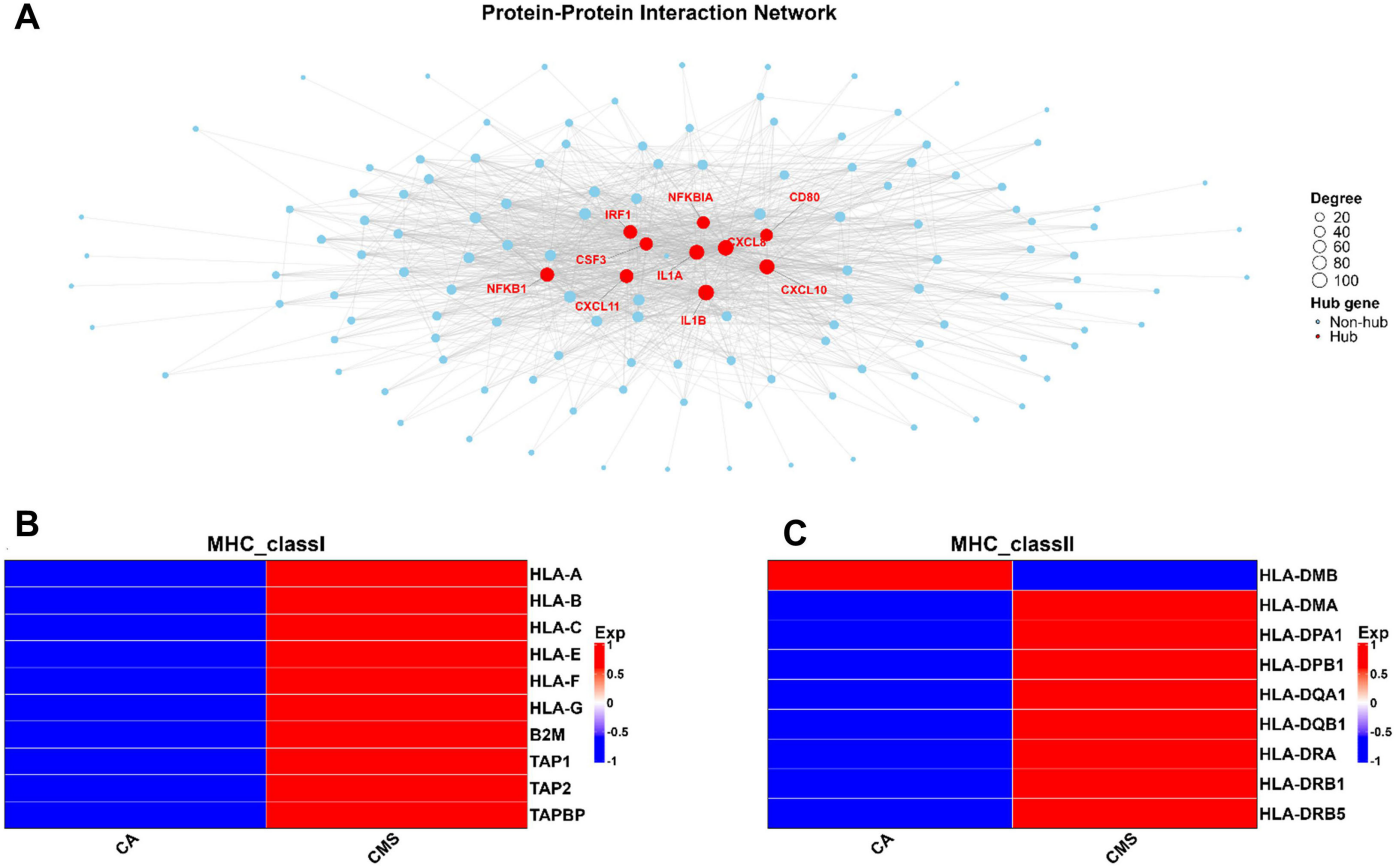
Protein-protein interaction (PPI) network and MHC gene expression in CMS-stimulated DCs. **(A)** Protein–protein interaction network constructed using the STRING database showing major hub genes (red nodes) among upregulated genes in CMS-treated DCs. Central hub genes include NFKB1, NFKBIA, IRF1, IL1A, IL1B, CXCL8, and CXCL10, which are key regulators of inflammatory and antiviral signaling. **(B)** Heatmap showing increased expression of MHC class I genes in CMS-treated DCs, indicating enhanced antigen processing and presentation *via* MHC-I pathway. **(C)** Heatmap showing upregulation of MHC class II genes, suggesting improved antigen presentation to CD4^+^ T cells. Together, these results demonstrate that CMS induces strong activation of antigen presentation machinery and pro-inflammatory transcriptional networks in human DCs. CA, cells alone.

Consistent with the pathway enrichment analysis revealing activation of TLR downstream signaling cascades, the PPI network identified the key hub genes involved in inflammatory and antiviral responses (**Figure 6A**). Using STRING-based interaction mapping, we observed densely interconnected networks, with NFKB1, NFKB1A, IL1A, IL1B, CXCL8, CXCL10, CXCL11, CSF3, IRF1, and CD80 emerging as central nodes with high degree values. These hub genes are known mediators of TLR-induced NF-κB, IRF, JAK-STAT, and cytokine-chemokine signaling pathways, supporting activation of both innate and adaptive immune effector programs in CMS-treated cells.

In parallel, differential expression analysis of major histocompatibility complex (MHC) genes revealed robust upregulation of both MHC class I and MHC class II genes in CMS-treated DCs compared with untreated cells (CA) (**Figures 6B, C**). The concurrent induction of pro-inflammatory hub genes and MHC-associated antigen processing pathways underscores the link between TLR-mediated innate signaling and enhanced antigen presentation capacity, thereby facilitating efficient adaptive immune priming.

### 3.7 TLR4/2 engagement is indispensable for CMS-induced signaling and activation of human APCs

**Figure 7:**
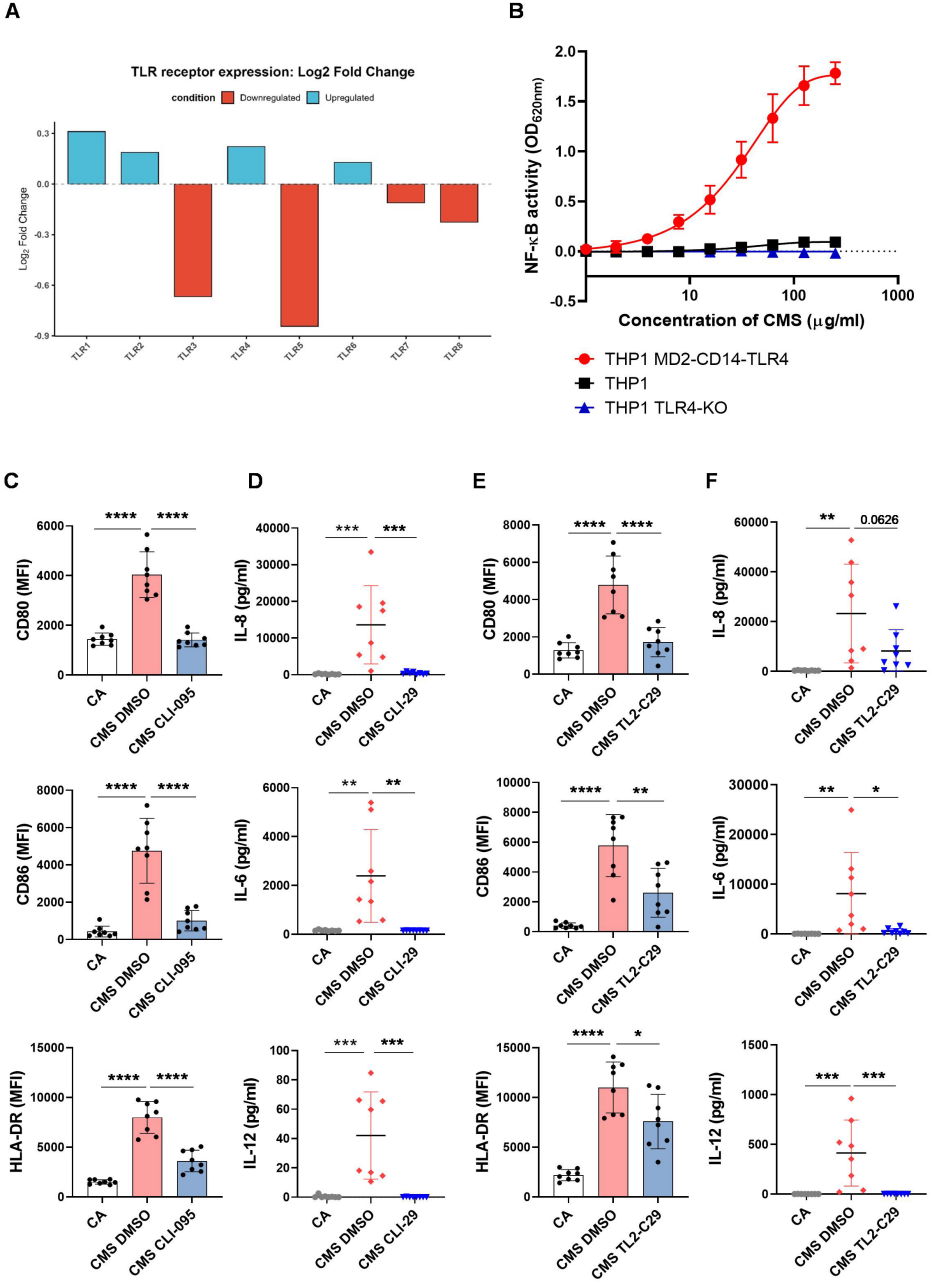
CMS activates DCs through TLR4 and TLR2-dependent mechanisms. **(A)** Heat map representation depicting the expression of different TLRs comparing unstimulated DCs vs CMS-stimulated DCs. **(B)** THP-1 human monocytic cell line stably expressing a secreted embryonic alkaline phosphatase (SEAP) reporter inducible by NF-kB was treated for 24 hrs with CMS at the indicated concentrations. The activation of NF-κB was assessed by measuring the activity of SEAP in the supernatant using QUANTI−Blue™ Solution. Results of THP1 MD2-CD14-TLR4, THP1 and THP1 KO-TLR4 are shown (mean ± SD, n=4). **(C, E)** Effect of CLI095 (TLR4 pathway inhibitor), or TL2-C29 (TLR2 pathway inhibitor), compared to DMSO (solvent control) on the expression of CMS-induced DC maturation markers. Scatter plots representing the mean ± SD (n = 8 donors) values of CD80, CD86, and HLA-DR are presented. **(D, F)** Inhibitory effect of CLI095 or TL2-C29 on the secretion (mean ± SD, n = 8) of IL-8, IL-6, and IL-12p70 (all in pg/ml) by CMS-stimulated DCs. *P < 0.05; **P < 0.01; ***P < 0.001; ****P < 0.0001 as analyzed by one-way ANOVA with Tukey’s multiple comparisons post-test **(C-F)**. CA, cells alone; DMSO, dimethyl sulfoxide; SD, standard deviation.

A strong upregulation of the TLR-dependent signaling pathway signatures in CMS-simulated DCs prompted us to investigate the functional role of the different TLRs in the stimulatory effect of CMS. A gene expression analysis of various TLRs in CMS-stimulated DCs compared to untreated controls (**Figure 7A**) revealed an upregulation of TLR1, TLR2, TLR4, and TLR6 transcripts.

To functionally validate the TLR-dependent NF-κB signaling by CMS, we resorted to the human monocytic THP-1 cell line stably expressing a secreted embryonic alkaline phosphatase reporter inducible by NF-κB. Although the THP-1 cells endogenously express various TLRs, it is significantly less sensitive to TLR4 agonists compared to primary human monocytes due to limited expression of the co-adaptors MD2 and CD14, which prompted us to include THP-1 cells overexpressing the MD2-CD14-TLR4 complex in our experiments. CMS induced a dose-dependent NF-κB activation in THP1 MD2-CD14-TLR4 cells; however, this activation was minimal in wild-type THP-1 cells and was completely abrogated in TLR4 knockout cells (**Figure 7B**). These results demonstrate that TLR4 engagement is essential for CMS-driven NF-κB signaling in APCs.

To corroborate these findings in primary cells, the role of TLR4 and TLR2 pathways was examined in human DCs using selective antagonists. Pre-treatment with CLI-095 (TLR4 inhibitor) or TL2-C29 (TLR2 inhibitor) markedly reduced CMS-induced expression of maturation markers and cytokine secretion compared with DMSO-treated controls (**Figures 7C–F**). In contrast, inhibition of TLR8 with CU-CPT9a had no significant effect (**Supplementary Figure 3**).

**Figure 8:**
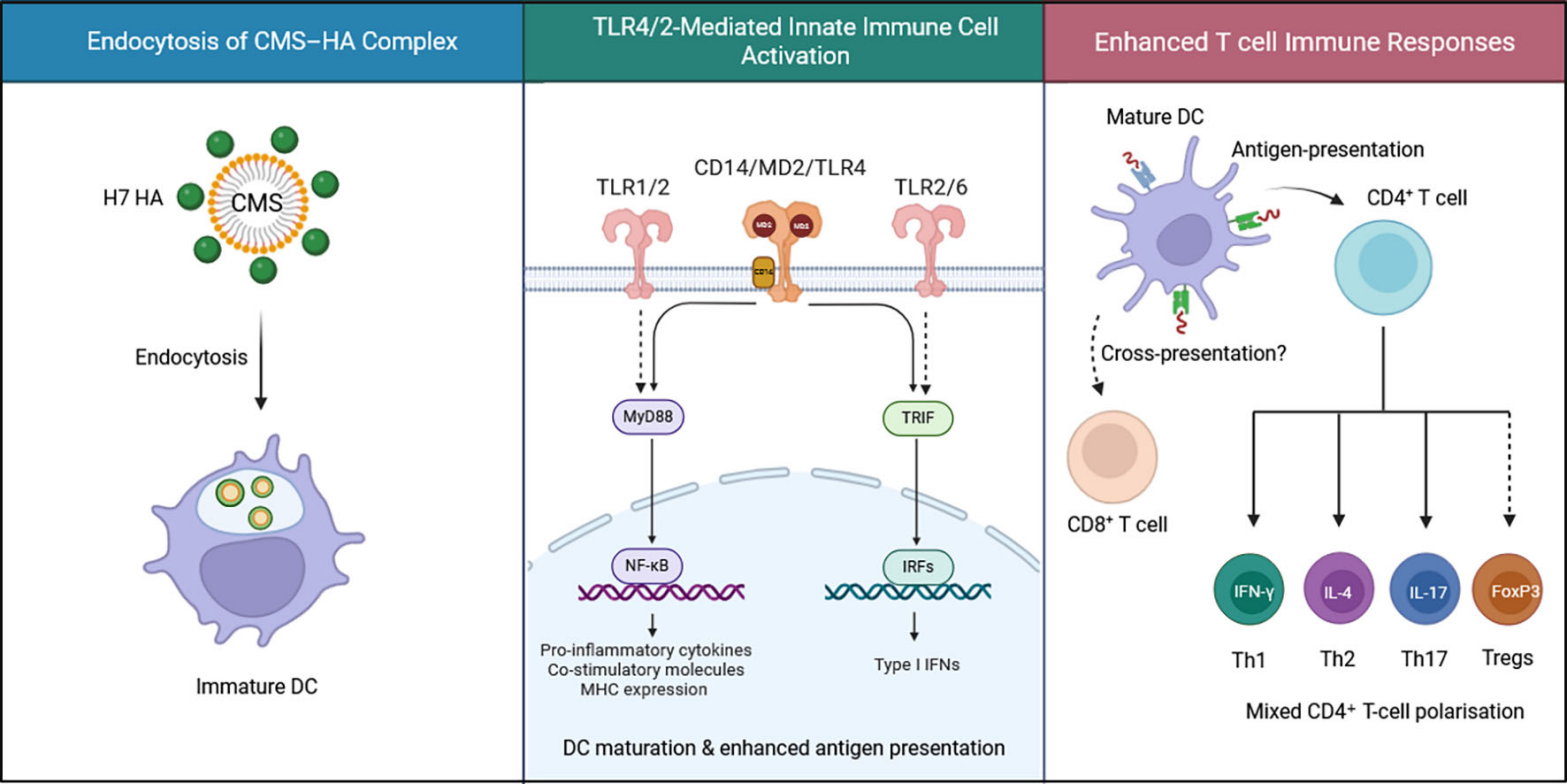
Proposed mechanisms of action of CMS. CMS associates with recombinant influenza hemagglutinin (HA) antigen and is endocytosed by antigen-presenting cells (APCs), such as dendritic cells. Following uptake, CMS engages pattern-recognition receptors, including TLR4–MD2–CD14 complexes and TLR2, triggering MyD88- and TRIF-dependent signaling pathways, which lead to the activation of NF-κB and IRF transcription factors, resulting in production of pro-inflammatory cytokines and type I interferons, as well as upregulation of co-stimulatory molecules and MHC expression. These events enhance antigen presentation capacity and support the induction of broad CD4^+^ T-helper responses together with increased CD8^+^ T-cell activation. Figure created at BioRender.com.

Collectively, these data identify TLR4 and TLR2-associated receptor complexes as critical mediators of CMS-induced activation in human APCs, bridging innate immune recognition with downstream pro-inflammatory and co-stimulatory signaling (**Figure 8**).

## 4 Discussion

The evolution of vaccination platforms from potent live attenuated/inactivated whole pathogens to safer and more economical subunit vaccines has taken a toll on their immunogenic potential, leading to the incorporation of adjuvants as an essential component of modern vaccines (38). The CMS adjuvant in the pipeline is a promising candidate to be added to the limited repertoire of safe and effective adjuvant systems approved for human use. The LiteVax™ formulation is a nano-emulsion composed of CMS, squalane, and Polysorbate 80. It forms droplets of approximately 80 nm. Its composition closely resembles widely used emulsion-based adjuvants such as MF59, which is a squalene-in-water emulsion stabilized by two non-ionic surfactants, Polysorbate 80 and Span 85.

However, it has been reported that MF59 adjuvant activity arises only from the complete formulation, as no single component exhibits intrinsic activity in isolation, either in an *in vivo* or *in vitro* setting (39). In contrast, CMS is a glycolipid similar to lipid A and monophosphoryl lipid A, and it shows inherent immunostimulatory properties. The LiteVax™ formulation combines these properties, where the emulsion serves as the delivery system of CMS, and CMS acts as the immunostimulatory agent, resulting in strong synergistic collaboration (25). Incorporating TLR agonists into oil-in-water emulsions has been demonstrated to enhance humoral and cellular immunity in preclinical studies (40–43), underscoring the translational potential of the concept.

CMS adjuvant formulation has been shown to augment immune responses to inactivated whole virus A/H5N1 and A/H7N9 vaccines in ferrets, toward a recombinant malaria antigen in rabbits (44), and more recently against the Receptor Binding Domain (RBD) spike protein antigens derived from SARS-CoV-2 in mice, with higher efficacy than comparator adjuvants tested in parallel (45). Preclinical toxicity assessments in ferrets and Phase I clinical trials have established the safety and tolerability of CMS with licensed inactivated seasonal influenza vaccines (46–48). CMS-adjuvanted low-dose vaccine (1/5^th^ antigen dose) gave comparable humoral and cell-mediated immunogenicity as the standard full-dose unadjuvanted vaccine, demonstrating efficient dose-sparing potential (46). A Phase Ib clinical trial with full doses of vaccine with or without CMS in younger and older, healthy adults revealed an acceptable safety profile in both cohorts and enhanced humoral immune responses in older adults, highlighting the potential of this adjuvant to counteract immune senescence (48).

In this report, we have investigated the immunological and molecular mechanisms underlying CMS adjuvanticity in primary human immune cells using various *in vitro* systems. The ability of CMS to enhance antigen-specific CD4^+^ and CD8^+^ T cell responses to H1N1 HA peptide pools in human PBMC assays recapitulates the strong humoral and cellular responses observed in clinical trials with low-dose adjuvanted influenza vaccine (46). While antigenic peptides alone failed to significantly expand CD8^+^ T cells, combining with the adjuvant substantially increased their frequency, pointing to the ability of CMS to promote cross-presentation and cytotoxic T lymphocyte activity. For a long time, the primary goal of vaccination strategies for respiratory viruses focused on the induction of neutralizing antibody responses. The recent severe SARS-CoV-2 variants shifted our attention to CD8^+^ T cells, with growing evidence showing that even the variants that escaped from neutralizing antibodies could not escape CD8^+^ T cells induced by mRNA and adenoviral vector vaccines (49). Influenza viruses are also typically known to escape from neutralizing antibodies *via* rapid mutations, and several studies have provided evidence for the protective role of cross-reactive CD8^+^ T cells against pandemic flu (50).

These observations suggest that adjuvants capable of eliciting robust CD8^+^ T cell responses might be particularly valuable for vaccines targeting highly variable respiratory pathogens. Our findings show that CMS drives strong antigen-specific CD4^+^ and CD8^+^ T cell responses *in vitro*, raising the possibility that CMS-adjuvanted recombinant influenza vaccines could enhance the magnitude and breadth of T cell immunity compared with antigen alone.

However, we did not assess *in vivo* protection or neutralizing antibody titers in this study. Therefore, any effect of CMS on vaccine efficacy, durability, or clinical benefit remains speculative. Future *in vivo* challenge studies, including detailed evaluation of neutralizing antibodies and cross-reactive CD8^+^ T cells, will be necessary to determine whether CMS adjuvantation can enhance and sustain protective immunity against seasonal and potentially pandemic influenza strains.

Further, the adjuvant capacity of CMS has been tested against recombinant flu HA protein antigens of the H7N9 strain in human APCs *in vitro*. Though an earlier study reported that recombinant HA proteins of H1N1 and H5N1 Influenza A viruses effectively promoted the activation and maturation of mouse myeloid DCs (42), experiments with human DC-based systems (DC-cell line MUTZ-3 and monocyte-derived DCs) (51) revealed the inefficacy of subunit vaccines of H5N1 to induce DC maturation, in contrast to whole inactivated virus (52). Consistent with these data, we demonstrated that H7N9 HA alone failed to activate human DCs. Combined with CMS, however, H7N9 HA elicited potent activation of human APCs, as evidenced by the enhanced expression of co-stimulatory molecules and release of cytokines. Fluorescence microscopy confirmed active endocytosis of the antigen-adjuvant complex by DCs, although CMS did not directly increase HA uptake. Though some adjuvants have the capacity to enhance antigen uptake by APCs, others, including CMS, mainly function through stimulating innate immunity and optimizing antigen presentation to generate robust adaptive immune responses (53).

Next-generation vaccine platforms utilize various advanced technologies to design antigens with precise epitope selection, aiming to elicit targeted cellular responses and to shape the T-cell repertoire. The choice of adjuvant also plays a pivotal role in this aspect, as different adjuvants have distinct effects on the polarization of T-cell responses. For example, adjuvants such as CpG, Adjuvant System (AS; AS01, AS03, AS04), or mRNA-lipid nanoparticles tend to promote strong Th1-oriented immunity, whereas alum or MF59 more commonly induce Th2-biased responses (54, 55). In our experiments, DCs stimulated with CMS supported the polarization of Th1, Th2, Th17, and FoxP3^+^ CD4^+^ T cell subsets using autologous total CD4^+^ T cells. This broad induction of T helper lineages demonstrates the capacity of CMS to engage multiple CD4^+^ differentiation pathways, potentially supporting both cytotoxic and humoral immunity. The induction of FoxP3^+^ regulatory T cells alongside effector T cells could be beneficial by limiting excessive inflammation and thereby reducing reactogenicity, which may enhance the safety profile of adjuvanted vaccines (56). On the other hand, vaccine antigen–specific Tregs can dampen responses to conserved epitopes (57), potentially complicating heterosubtypic protection. However, FoxP3 expression in human CD4^+^ T cells at early stages of activation can also reflect transient activation of conventional T cells rather than stable regulatory T cells (58), and functional suppressive activity of the FoxP3^+^ CD4^+^ T cells population was not assessed in this study. Thus, while CMS clearly promotes a diverse phenotypic T helper response, the precise regulatory capacity of the FoxP3^+^ CD4^+^ T cell-and its biological significance in shaping vaccine-induced immunity-will require further functional and longitudinal investigation.

Despite strong evidence for the induction of immune responses by CMS in *in vivo* and *in vitro* systems, the receptors and the downstream signaling pathways involved in this process were not described. TLRs play a major role in innate immunity by responding to various pathogen-derived ligands, thereby activating various pro-inflammatory signaling pathways (59). Our transcriptomic profiling showed that stimulation of human DCs with CMS led to strong activation of innate immune signaling pathways that result in the upregulation of NF-κB, IL-1, MAPK, JAK-STAT, and Interferon pathways. Engagement of TLR-2/4 triggers MyD88/TRIF pathways that activate NF-κB and MAPKs, leading to rapid transcription of pro-inflammatory cytokines and co-stimulatory molecules as observed in our analysis. The concurrent induction of JAK-STAT and interferon pathways suggests that CMS also engages in the induction of interferons (**Supplementary Figure 4**). Thus, CMS appears to mimic key infection signals and play a key role in DC maturation by activating pro-inflammatory cytokines and co-stimulatory molecules essential for initiating adaptive immune responses.

Our RNA-seq analysis is based on a relatively small cohort (n = 3 donors per group), which represents a limitation of the study. This dataset was however generated as a mechanistic discovery cohort rather than a population-level study, and the transcriptomic results are therefore interpreted as hypothesis-generating and supported by complementary experimental and functional evidence presented in this report. Furthermore, principal component analysis (PCA) revealed clear separation between CMS and control samples along PC1, which explained most of the variance, indicating that biological condition was the dominant driver (**Supplementary Figure 5**). The within-group dispersion observed along PC2 likely reflects inter-donor variability rather than technical batch effects, particularly given the small sample size and uniform experimental processing. Consequently, no batch correction was applied to avoid removal of biological signal. Future studies incorporating larger and independent experimental cohorts would be valuable to further validate and extend these observations.

Key hub genes identified in the protein-protein interaction network (**Figure 6A**) further highlighted the immunostimulatory effects of CMS. These genes play central role in DC functions: NFKB1 - a master regulator of inflammatory genes (60); IL-1B encodes for IL-1β, a potent pro-inflammatory cytokine produced by activated DCs (61); CXCL10, an interferon inducible chemokine recruits CXCR3^+^ effector T cells and is characteristic of Th1-type responses (62); IRF1 drives the antigen presentation by amplifying the MHC expression (63), which is consistent with our observation of increased MHC-1/II expression in CMS-treated DCs. Upregulation of both the MHC classes substantiates our *in vitro* data showing that CMS enhances the ability of DCs to present antigens to both CD4^+^ and CD8^+^ T cells. Collectively, the enhanced antigen presentation signature underscores CMS’s ability to strengthen the innate and adaptive interface, which is a prerequisite for robust vaccine responses (**Figure 8**).

We have previously shown that the monosulphate group in CMS is indispensable for the adjuvanticity of CMS in DCs (26). Some endogenous ligands containing a sulfate group, namely heparan sulfate and sulphatides, are reported to act as TLR4 agonists (64, 65). The binding of the sulfate group with TLR4 is also confirmed by the structural analysis of the binding of sulfatide with TLR4 (65), which activates MyD88 and TRIF-dependent signaling (66). Our experiments with THP-1 reporter assays demonstrated the activation of the NF-κB pathway in response to CMS, especially with the overexpression of the MD2-CD14-TLR4 receptor complex. Similar experiments were carried out previously with HEK reporter cells overexpressing the TLR4 receptor complex, which did not show any reporter activity with CMS stimulation (44), indicating that endocytic processing by APCs might be a prerequisite for TLR engagement by CMS. Functional blocking studies with TLR antagonists confirmed the role of TLR2 alongside TLR4 in CMS-induced activation of DCs. Although transcriptomic analysis revealed upregulation of TLR1, TLR2, and TLR6, the functional involvement of specific TLR2 heterodimers (TLR1/2 versus TLR2/6) remains unresolved. Heterodimer-specific neutralization assays will be required to address this question in future studies.

TLR4 and TLR2 both promote DC maturation by upregulating expression of CD80, CD86, and MHC-I/II. However, due to the TRIF pathway activated by TLR4, it gives a strong antiviral response, pushing DC toward a Th1 immune profile and enhancing cross presentation with CD8^+^ T cells (67). While TLR2 gives a balanced immune response without strongly polarizing between Th1 and Th2. Experimental data have shown that compared to LPS-treated DC, TLR2 agonists such as SUP1 boost DC antigen presentation to CD4^+^ T cells and enhance memory cell formation against the antigen (68). Moreover, TLR4 leads to sustained IL-10 production as the TRIF pathway has been shown to stabilize the IL-10 mRNA (69). Thus, in vaccine terms, concomitant engagement of TLR4 and 2 becomes more beneficial. These findings position CMS as a promising, compatible adjuvant that efficiently activates DCs *via* TLR-signaling and interferon pathways, augmenting cytokine and co-stimulatory molecule expression alongside antigen processing gene upregulation to mount a robust interface between innate sensing and adaptive immunity.

Our current study is limited to evaluating CMS-induced responses in PBMC-derived immune cell populations from healthy donors, which does not fully recapitulate the spatial and cellular complexity of immune activation *in vivo*. While these *in vitro* data provide initial mechanistic insight into early innate sensing and downstream activation, validation in relevant *in vivo* challenge models, such as mice or ferrets, will be essential to determine whether the CMS adjuvant enhances protective immunity and promotes robust neutralizing antibody responses. Such models will enable assessment of antigen-presenting cell activation, trafficking to draining lymph nodes, and the induction of germinal center reactions that support durable humoral immunity. Furthermore, single-cell transcriptomic profiling of PBMCs from clinical cohorts could provide an unbiased view of responsive innate subsets and T-cell polarization states. Together, these complementary approaches will enable validation of the primary target cell populations and elucidation of the receptor-mediated mechanisms of CMS within a more physiologically relevant setting with larger sample sizes, thereby strengthening the translational relevance of our findings and informing the rational optimization of CMS-based vaccine formulations.

In conclusion, this study provides proof of concept for the adjuvant activity of CMS against influenza HA antigens in *in vitro* immune cell systems. CMS induces strong antigen-specific T cell responses, especially CD8^+^ T cell responses to HA antigens in human PBMCs. Furthermore, CMS enhances the immunostimulatory potential of recombinant H7N9 HA antigen on APCs and induces a mixed effector T cell polarization. Mechanistically, CMS activates the TLR4 and TLR2-associated signaling, leading to cytokine/chemokine secretion that promotes innate immune cell recruitment, activation, and antigen presentation to T cells, thereby supporting strong humoral and cellular immune responses. Further evaluation of the adjuvant antigen formulation in appropriate animal models of various age groups (70) is warranted to assess the generation of neutralizing antibodies against the recombinant vaccine antigens and protection against influenza infection. Our results underscore the potential of the CMS recombinant HA antigen formulation for further clinical development and help in the design of effective novel vaccine adjuvant formulations to protect the vulnerable population from influenza-related complications. 

## Data Availability

The datasets presented in this study can be found in online repositories. The names of the repository/repositories and accession number(s) can be found below: GSE318332 (GEO).
