# Supplementary material for: Carbohydrate fatty acid monosulphate ester adjuvant enhances the immunogenicity of influenza antigens via TLR4/2-dependent mechanisms
Source: Front Immunol. 2026 Mar 4;17:1787181. doi: 10.3389/fimmu.2026.1787181 (PMC12995642; doi:10.3389/fimmu.2026.1787181)
Supplement: Supplementary file 1 [file DataSheet1.pdf]

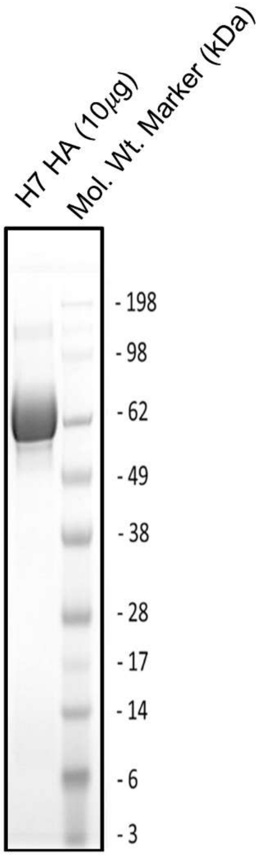

Figure S1: Coomassie-stained SDS-PAGE analysis of purified H7N9 HA.

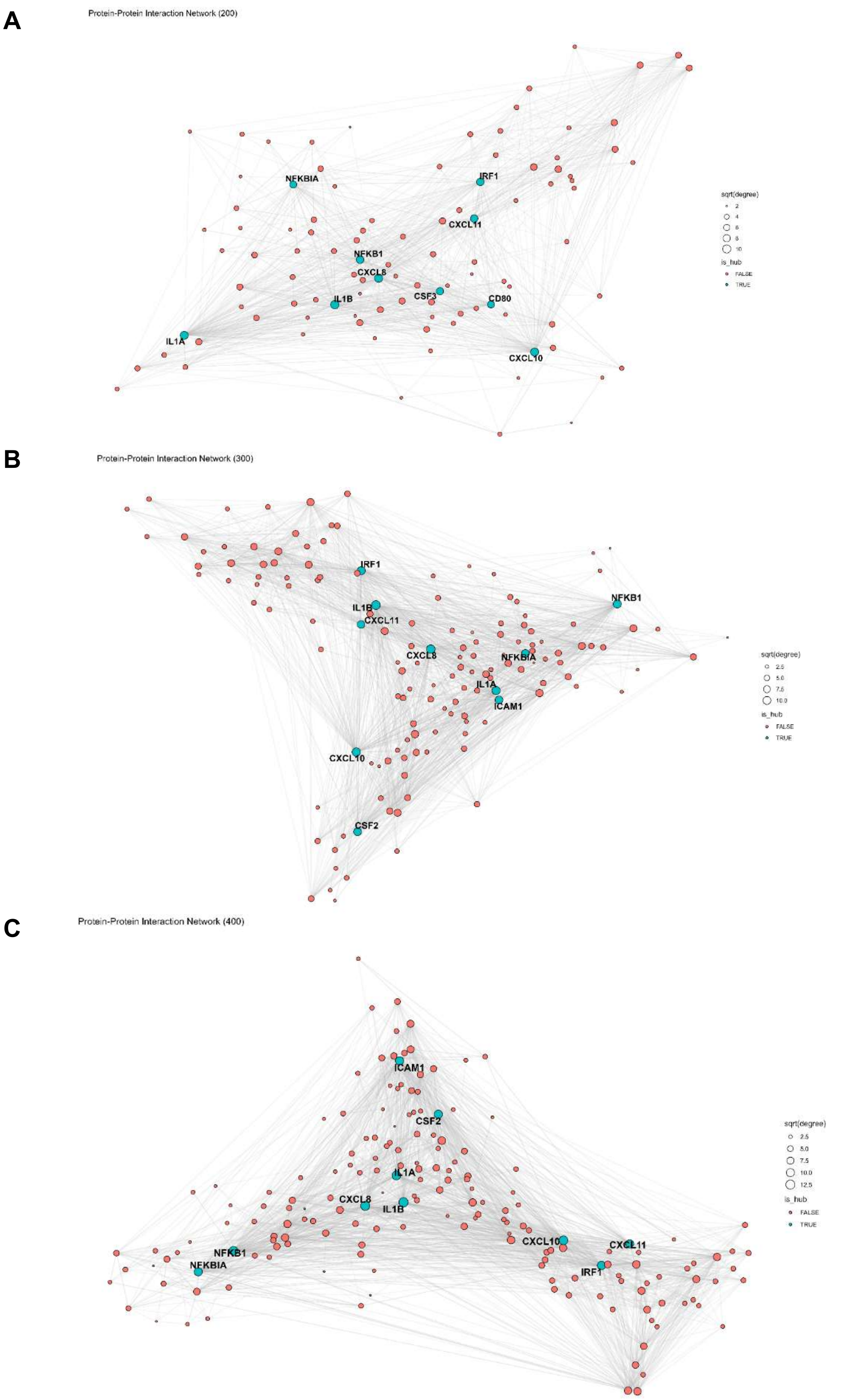

Figure S2: Protein–protein interaction (PPI) network of differentially expressed genes**.** The PPI network was constructed using the top 200 (**A**), 300 (**B**) or 400 (**C**) differentially expressed genes (DEGs) based on STRING database interactions. Node size reflects connectivity (degree), and hub proteins are highlighted and labelled.

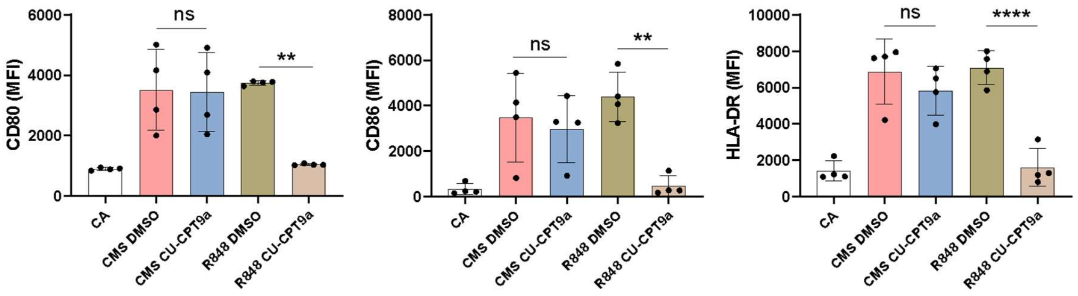

Figure S3: Effect of CU-CPT9a (TLR8 pathway inhibitor compared to DMSO (solvent control) on the expression of CMS-induced dendritic cell maturation markers (mean ± SD, n = 4 donors). R848 (TLR8 agonist) was used as a positive control. Expression (median fluorescence intensities, MFI) of CD80, CD86, and HLA-DR are presented. Statistics are **$P < 0.01$; ****$P < 0.0001$; *ns*, not significant as determined by one-way ANOVA with Tukey's multiple comparisons post-test. Abbreviation: CA, cells alone; DMSO, dimethyl sulfoxide; SD, standard deviation.

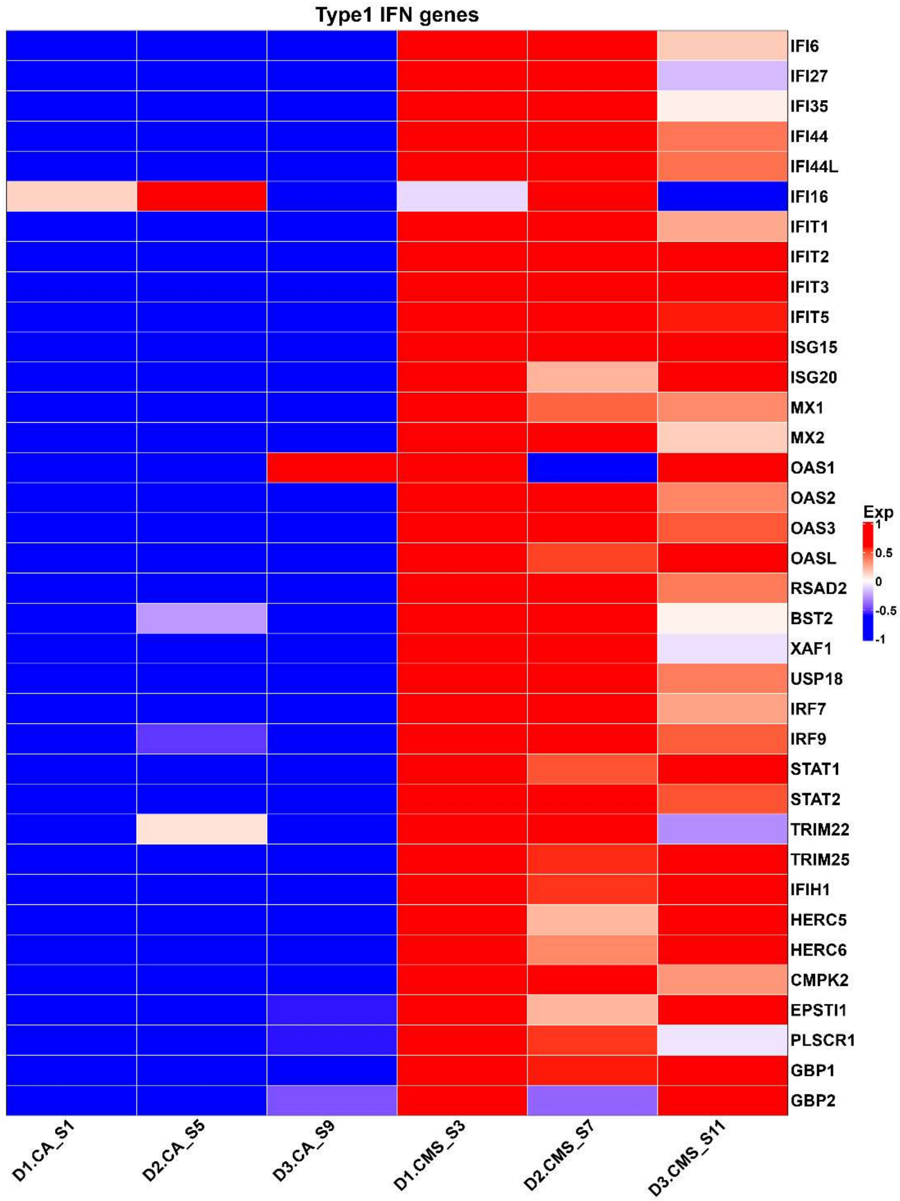

Figure S4: Type 1 Interferon gene expression in CMS-stimulated DCs compared to untreated cells.

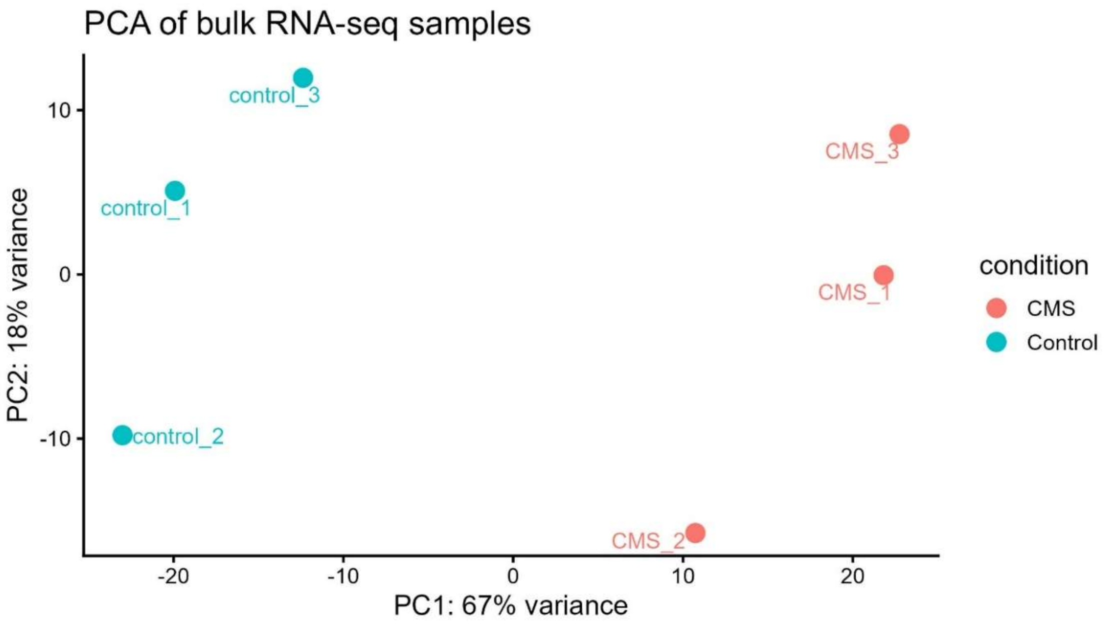

Figure S5: Principal component analysis (PCA) of bulk RNA-seq samples. PCA was performed on variance-stabilized gene expression values. Each point represents an individual donor sample, coloured by condition (Control and CMS).
